# Intake of flavonoids from *Astragalus membranaceus* ameliorated brain impairment in diabetic mice via modulating brain-gut axis

**DOI:** 10.1186/s13020-022-00578-8

**Published:** 2022-02-12

**Authors:** Xuling Li, Tingting Zhao, Junling Gu, Zhe Wang, Jing Lin, Rushang Wang, Tingting Duan, Zhenghai Li, Ruixue Dong, Weiming Wang, Kin-Fong Hong, Zhilong Liu, Wenbo Huang, Dingkun Gui, Hua Zhou, Youhua Xu

**Affiliations:** 1 Faculty of Chinese Medicine, State Key Laboratory of Quality Research in Chinese Medicine, Macau University of Science and Technology, Taipa, Macao People’s Republic of China; 2 Guangdong Provincial Hospital of Chinese Medicine-Zhuhai Hospital, Zhuhai, Guangdong China; 3 Institute of Consun Co. for Chinese Medicine in Kidney Diseases, Guangdong Consun Pharmaceutical Group, Dongpeng Road 71, Guangzhou, China; 4 School of Pharmacy, State Key Laboratory of Quality Research in Chinese Medicine, Macau University of Science and Technology, Taipa, Macao, People’s Republic of China; 5 Department of Endocrinology, Zhuhai Hospital of Integrated Traditional Chinese and Western Medicine, Zhuhai, People’s Republic of China; 6 Department of Nephrology, Shanghai Jiao Tong University Affiliated to Sixth People’s Hospital, Shanghai, People’s Republic of China; 7 Zhuhai MUST Science and Technology Research Institute, Hengqin, Zhuhai, People’s Republic of China

**Keywords:** *Astragalus membranaceus*, Brain, Diabetes, Gut, Microbiota

## Abstract

**Background:**

Brain impairment is one of a major complication of diabetes. Dietary flavonoids have been recommended to prevent brain damage. *Astragalus membranaceus* is a herbal medicine commonly used to relieve the complications of diabetes. Flavonoids is one of the major ingredients of *Astragalus membranaceus,* but its function and mechanism on diabetic encepholopathy is still unknown.

**Methods:**

Type 2 diabetes mellitus (T2DM) model was induced by high fat diet and STZ in C57BL/6J mice, and BEnd.3 and HT22 cell lines were applied in the in vitro study. Quality of flavonoids was evaluated by LC–MS/MS. Differential expressed proteins in the hippocampus were evaluated by proteomics; influence of the flavonoids on composition of gut microbiota was analyzed by metagenomics. Mechanism of the flavonoids on diabetic encepholopathy was analyzed by Q-PCR, Western Blot, and multi-immunological methods et al.

**Results:**

We found that flavonoids from *Astragalus membranaceus* (TFA) significantly ameliorated brain damage by modulating gut-microbiota-brain axis: TFA oral administration decreased fasting blood glucose and food intake, repaired blood brain barrier, protected hippocampus synaptic function; improved hippocampus mitochondrial biosynthesis and energy metabolism; and enriched the intestinal microbiome in high fat diet/STZ-induced diabetic mice. In the in vitro study, we found TFA increased viability of HT22 cells and preserved gut barrier integrity in CaCO_2_ monocellular layer, and PGC1α/AMPK pathway participated in this process.

**Conclusion:**

Our findings demonstrated that flavonoids from *Astragalus membranaceus* ameliorated brain impairment, and its modulation on gut-brain axis plays a pivotal role. Our present study provided an alternative solution on preventing and treating diabetic cognition impairment.

## Introduction

Type 2 Diabetes mellitus (T2DM), an aging related disease, has been a public health challenge world-wide. Cognitive decline is a serious complication of T2DM [1, 2], but specific mechanism is still unknown. Previous studies indicated that diabetes-induced cognitive impairment is accompanied with amyloid β-protein (Aβ) aggregation, tau hyperphosphorylation, glucose absorption decline, neurotransmitter metabolism disorders, and mitochondrial dysfunction [3–5]. Recent findings suggest that gut microbiota as well as the co-metabolism between microbiota and the organism also play an important role in the disease development [6–8]. Strategies on modulating metabolism within gut lumen have witnessed positive effects against brain dysfunction [9–11], but direct evidence and mechanism is few.

Food-borne flavonoids have been recommended for the prevention and treatment of cognitive impairment [12, 13]. *Astragalus membranaceus (Huang Qi* in Chinese*)* is a traditional herbal medicine that has been applied for treating diabetes for hundreds of years. Flavonoids exist widely in the plant kingdom, and are more abundant in the plants of the *Fabaceae*, *Compositae*, *Lamiaceae* and *Rutaceae*, it is an active ingredient of many Chinese herbal medicines such as *Sophora Japonica*, *Astragalus*, *Tangerine Peel*, *Pueraria*, *Wild Chrysanthemum*, *Milk Thistle*, *Ginkgo Biloba* and so on. According to statistics, *Astragalus membranaceus* is the most frequently used one among all anti-diabetic Chinese medicines. One of the most important components within *Astragalus membranaceus* is flavonoid; therefore, their pharmacological influence on diabetic complications deserves to be deeply investigated.

So far, about 40 kinds of flavonoids have been isolated from *Astragalus* plants, mainly including kaempferol, quercetin, Rhamnetin, rhamnetin glycosides, isorhamnetin, verbascoflavones, verbascoside glycosides, formononetin, and genistein, et al. [14]. Calycosin, Ononin, Calycosin-7-O-beta-d-glucoside, and Formononetin are found to be the main chemical components of flavonoids from *Astragalus membranaceus*. Among them, isoflavones and isoflavone glycosides are the main active components contributing to their antioxidant capacity. It was reported that calycosin and formononetin can prevent nerve damage and improve brain function in diabetic mice via increasing glycogen metabolism and decreasing oxidative stress [15–17], and quercetin can effectively clear Aβ and ameliorate cognitive impairment [18]. Previously, we found calycosin can reduce generation of advanced glycation end products (AGEs), modulate glucose metabolism within hepatocytes, inhibit vascular endothelial cell damage, and exert beneficial effects against development of diabetes [19, 20]. However, exact effects and mechanism of flavonoids on diabetic encephalopathy is still unknown.

Gut-brain crosstalk is recently believed to play a pivotal role in modulating a series of pathophysiological process, including diabetes. It mainly include brain, brain-derived neutotrophic factors, gut microbes, gut and its secreted neuro-peptides, and autonomic nervous system [21]. A two-way communication between gut and brain makes it possible to modulate brain dysfunction via gut-axis. Previous studies found that flavonoids can modulate composition of gut microbiota [22–25], and promote co-metabolism between microbiota and the organism. Converging evidences above, we postulated that flavonoids from *Astragalus membranaceus* may possess protective effects against diabetes-induced brain damage via gut-brain axis. For this aim, effects and mechanism of TFA on diabetic-brain damage was investigated both in vivo and in vitro.

## Materials and methods

### Materials

TFA and Metformin were bought from Pusi Biotecnology (Chengdu, Sichuan, China, purity: ≥ 90.0%) and GBCBIO technology (Guangzhou, Guangdong, China), respectively. Detection kits for AGEs, 5-hydroxytryptamine (serotonin, 5-HT), amyloid β peptide 1–42, amyloid β peptide 1–40, and lipopolysaccharide (LPS) were purchased from Nanjing Jiancheng Bioengineering Institute (Nanjing, Jiangsu, China). Kits for interleukin-1β (IL-1β), tumor necrosis factor-α (TNF-α), interleukin-6 (IL-6), and brain derived neurotrophic factor (BDNF) were derived from Solarbio (Beijing, China). Primary-antibodies including postsynaptic density protein 95 (PSD95), Synapsin I a/b, estrogen receptor β (ERβ), heat shock proteins 60 (HSP60), heat shock proteins 70 (HSP70), and caseinolytic protease (CLPP) were purchased from Santa Cruz (Dallas, TX, USA); phospho-AMPK, AMPK, CREB and phospho-CREB were from Cell Signaling (USA); advanced glycosylation end product-specific receptor (RAGE), Lon protease (LONP1) and β-actin were from Proteintech (Chicago, USA); PGC-1α was from Bioss (Beijing, China); ZO-1, Claudin 5, and Occludin were from Invitrogen (California, USA); GABA A Receptor beta 1 (GABAR β), glucagon-like peptide 1 (GLP-1) were obtained from Affinity Biosciences (USA). Other materials and reagents were from commercial sources.

### Animals

It have been demonstrated that high-fat diet impairs brain function [26, 27]. To this end, we applied STZ combined with high-fat diet to induce diabetic brain dysfunction model in the present study. Eight to ten weeks old male C57BL/6J mice were bought from Guangdong medial laboratory animal center and housed with the standard condition of 12 h light and 12 h dark cycle. All animal care and operations were according to the guidelines of the U.S. National Institutes of Health in the care and use of Laboratory Animals (NIH publication No. 85-23, revised 1996) and approved by Macau University of Science and Technology. Mice were firstly divided into natural control (NC) group and high-fat-diet (HFD) group. NC mice were given with normal diet, in kcal: 19% proteins, 68% carbohydrate, and 13% fat. Mice in HFD group were given with high fat diet, in kcal: 15% proteins, 43% carbohydrate, and 42% fat; 8 weeks later, these mice were intraperitoneal injected with STZ (50 mg/kg d) for 5 days to induce T2DM, and mice with the fasting glucose above 11.1 mmol/l after 12 h fasting or 2 h intraperitoneal glucose tolerance test (IPGTT) ≥ 11.1 mmol/l were included for thereafter study. The included diabetic mice were randomly divided into groups as follows: (1) T2DM group (HFD, n = 4); (2) High-dose TFA group (TFA-H group, HFD, TFA 50 mg/kg d, n = 6); (3) Medium-dose TFA group (TFA-M group, HFD, TFA 25 mg/kg d, n = 7); (4) Low-dose TFA group (TFA-L group, HFD, TFA 5 mg/kg d, n = 6); and (5) positive control group (HFD, metformin, 0.15 g/kg d, n = 4). All drugs were orally administrated for 16 weeks. The body weight, fasting glucose and food intake were recorded every one or two weeks. At the end of the experiment, mice were killed by cervical dislocation method; the serum, fecal, brain and gut tissues were collected for further study.

### Hematoxylin–eosin (H&E) staining and immunohistochemistry (IHC)

The fresh whole brain tissue and intestine tissues were fixed with 4% paraformaldehyde. Two days later, the tissues were made into paraffin block. For H&E staining, the sections were stained according to standard protocol. For IHC, the sections were incubated with primary antibodies as indicated overnight at 4 °C. After three times’ washing with phosphate buffer saline (PBS), the sections were exposed to secondary antibody for 1 h at room temperature. After DAB coloration and redye nucleus by hematoxylin, the sections were sealed with neutral resin. Results of H&E and IHC were observed by Olympus microscope and analyzed by Image-J software.

### ELISA

Mice were administrated with 5% pentobarbital sodium by intraperitoneal injection and blood was collected by ophthalmectomy. Blood was centrifuged at 3,000 rpm for 15 min, and the supernatant was collected as serum. Fresh brain was homogenized by homogenizer, and was centrifuged at 3000 rpm for 20 min, supernatant was finally collected. The concentration of BDNF, AGEs, LPS, Aβ (1–40), Aβ (1–42), 5-HT, IL-1β, TNF-α and IL-6 were tested according to the manufacturers’ protocols.

### Mitochondrial DNA extraction

Mitochondrial and genomic DNA within hippocampus tissues were extracted by QIAamp^®^ DNA Micro Kit according to protocol supplied by the supplier. DNA concentration of the samples was measured with NanoDrop ND 2000 Ultra-micro spectrophotometer, and quantified to 45 ng/µl for subsequent Q-PCR.

### Q-PCR

Total RNA from the sample was isolated by TRIzol reagent. RNA concentration was determined by NanoDrop ND 2000 Ultra-micro spectrophotometer. RNA was reverse-transcripted into cDNA by PrimerScript™ RT reagent Kit (Perfect Real Time) according to the protocol supplied. Q-PCR reaction was conducted according to TB Green™ Premix Ex Taq™ II (Tli RNaseH Plus) protocol supplied by the supplier. The reaction system for 96 well plates is as follow: 10 µl of TB Green™ Premix Ex Taq™ II (Tli RNaseH Plus) (2×), 0.8 µl of PCR Forward Primer (10 µM), 0.8 µl of PCR Reverse Primer (10 µM), 2 µl of DNA sample (< 100 ng), and 6.4 µl of ddH_2_O. Q-PCR reaction was took place on LightCycler 480 System according to the supplier’s protocol. Primer sequences used in Q-PCR assay are listed in Table 1.

**Table 1:** Primers used for analysis of mtDNA copy number or HT22 cells gene expression by Q-PCR

Gene	Forward primers (5′ to 3′)	Reverse primers (5′ to 3′)
β-actin	CTACCTCATGAAGATCCTGACC	CACAGCTTCTCTTTGATGTCAC
RAGE	CTACCTTCTCCTGCAGTTTCAG	CATCCTTTATCCAGTGGACCTG
BDNF	GATGCCGCAAACATGTCTATG A	TAATACTGTCACACACGCTCAGCTC
Cox3	TTTGCAGGATTCTTCTGAGC	TGAGCTCATGTAATTGAAACACC
Ndufv1	CTTCCCCACTGGCCTCAAGC	CCAAAACCCAGTGATCCAGC

β-actin, beta-actin; RAGE, advanced glycosylation end product-specific receptor; BDNF, brain derived neurotrophic factor; Cox3, cytochrome c oxidase subunit 1; Ndufv1, NADH dehydrogenase (ubiquinone) flavoprotein 1

### Western blot

The proteins were extracted by RIPA. Total proteins were separated by SDS polyacrylamide gel (SDS-PAGE), and then transferred to PVDF membrane by wet transfer apparatus. Membranes were incubated with primary antibodies indicated at 4 °C overnight. After three times’ washing with TBST, the membranes were exposed to secondly antibody for 1 h at room temperature. Bands were developed by ETC, and relative expression of proteins were analyzed by Image-J software according to the density of bands.

### Proteomic analysis of hippocampus tissues

Fresh brain tissues were suspended by protein extraction buffer and then ultrasonicated with Fisher 550 Sonic Dismembrator (Pittsburgh, PA, USA). Protein homogenates were centrifuged at 12,000*g* at 4 °C for 20 min, and the samples were mixed with 10 mM dithiothreitol (DTT) and further incubated at 55 °C for 30 min. After that, samples were incubated with 25 mM iodoacetamide (IAA) at dark environment for 1 h and digested with the buffer concluding 1.0 M urea and trypsin (1:100 w/w) at 37 °C for 14 h. Salt was removed from the peptides by centrifuging at 12,000*g* for 15 min using a reversed-phase column. Then the peptides were labeled by TMT 6-plex reagents. After that, peptides were separated and analyzed by LC–MS/MS. The peptides were compared with UniProt mouse FASTA database using Proteome Discoverer 2.1 software. Protein quantification were detected by reporter ion intensities of every peptide. Ratio ≥ 1.1 or ≤ 0.91 were set as increase or decrease thresholds, respectively. The proteins significance analysis was set at p < 0.05 using Perseus statistical software. The functional enrichment analysis of Gene Ontology for biological process was realized using DAVID Bioinformatics Resources 6.8. The oxidation phosphorylation pathway was analyzed by Cytoscape 3.7.1 software and Kyoto Encyclopedia of Genes and Genomes (KEGG) path database. Protein co-expression network was analysis by STRING and Cytoscape 3.7.1.

### 16S/18S rRNA gut microbial sequencing

DNA from fecal samples were extracted using CTAB/SDS method. After quality identification, the DNA was quantificated to 1 ng/μl before PCR. The PCR Primers were: 16S V4: 515F-806R, 18S V4: 528F-706R, 18S V9: 1380F-1510R, ITS1: ITS5-1737F, ITS2-2043R, ITS2: ITS3-2024F, ITS4-2409R. PCR situation was set as follows: 98 °C, Pre-degeneration; 98 °C, 10 s; 50 °C, 30 s; 72 °C, 30 s; 30 cycles; lasting 72 °C for 5 min. PCR-free library was constructed on Illumina Nova and then Paired-End sequencing was performed. Sequence was clustered as Operational Taxonomic Units (OTUs) with 97% identity and compared with Silva132 [28]. Metastat test was used in Heatmap analysis. Principal Co-ordinates Analysis (PCoA) and Non-Metric Multi-Dimensional Scaling (NMDS) were applied in Beta Diversity analysis.

### Cell culture and MTT assay

BEnd.3 and HT22 cell lines were respectively cultured in DMEM supplemented with 10% FBS, 100 mg/ml streptomycin, and 100 U/ml penicillin at 37 °C and 5% CO_2_. For MTT assay, the cell was cultured in a 96-well cell culture plate. Different concentrations of TFA (1–20 μg/ml) or AGEs (50–800 μg/ml) were added to the culture system. Twenty-four or 48 h later, the cells were cultured with MTT (0.5 mg/ml) for 4 h in the dark. DMSO was added and the cell viability were observed on 490 nm by enzyme-labeled instrument.

### Immunofluorescence

The cells were cultured on the glass slides within 12-well cell culture plate. At exponential phase, cells were fixed by 4% paraformldehyde. After being blocked with 5% BSA, cells were incubated with primary antibodies as indicated overnight at 4 °C and then gently washed with PBS for three times. Secondary antibodies were co-cultured with the cells at room temperature for 1 h. DAPI was applied to stain the nucleus. Cell morphology was observed by confocal laser scanning microscopy (Leica TCS SP8, Germany) and the fluorescence intensity were determined by Image J software.

### Gut barrier integrity test in vitro

In vitro gut barrier model was constructed according to previous reports [29, 30]. In general, 0.3 × 10^4^ CaCO_2_ cells suspension was seeded on the transwell polycarbonate membrane and cultured at 37 °C, 5% CO_2_ for 21 days to construct a mono-cellular barrier model. LPS was used to induce the mono-cellular barrier dysfunction, and either TFA or MET was applied to observe their therapeutic effects. Forty-eight hours later, fluorescein isothiocyanate (FITC)-dextran (70 kDa, 2 mg/ml) was added into the apical chamber, thereafter, 50 μl media in the basolateral chamber was collected for every 30 min to evaluate the barrier permeability by SpectraMax Paradigm Multi-Mode Detection Platform.

### Statistical analysis

Except for data of proteomics and intestine microbiome, the other data was expressed as mean ± SEM. Significant difference between-groups were determent by Column analysis *t*-test, and one-way ANOVA analysis was applied to compare difference among groups by GraphPad Prism 8. Significance was set at *p* < 0.05 or less.

## Results

### TFA decreased blood glucose and energy intake in T2DM animals

**Fig. 1:**
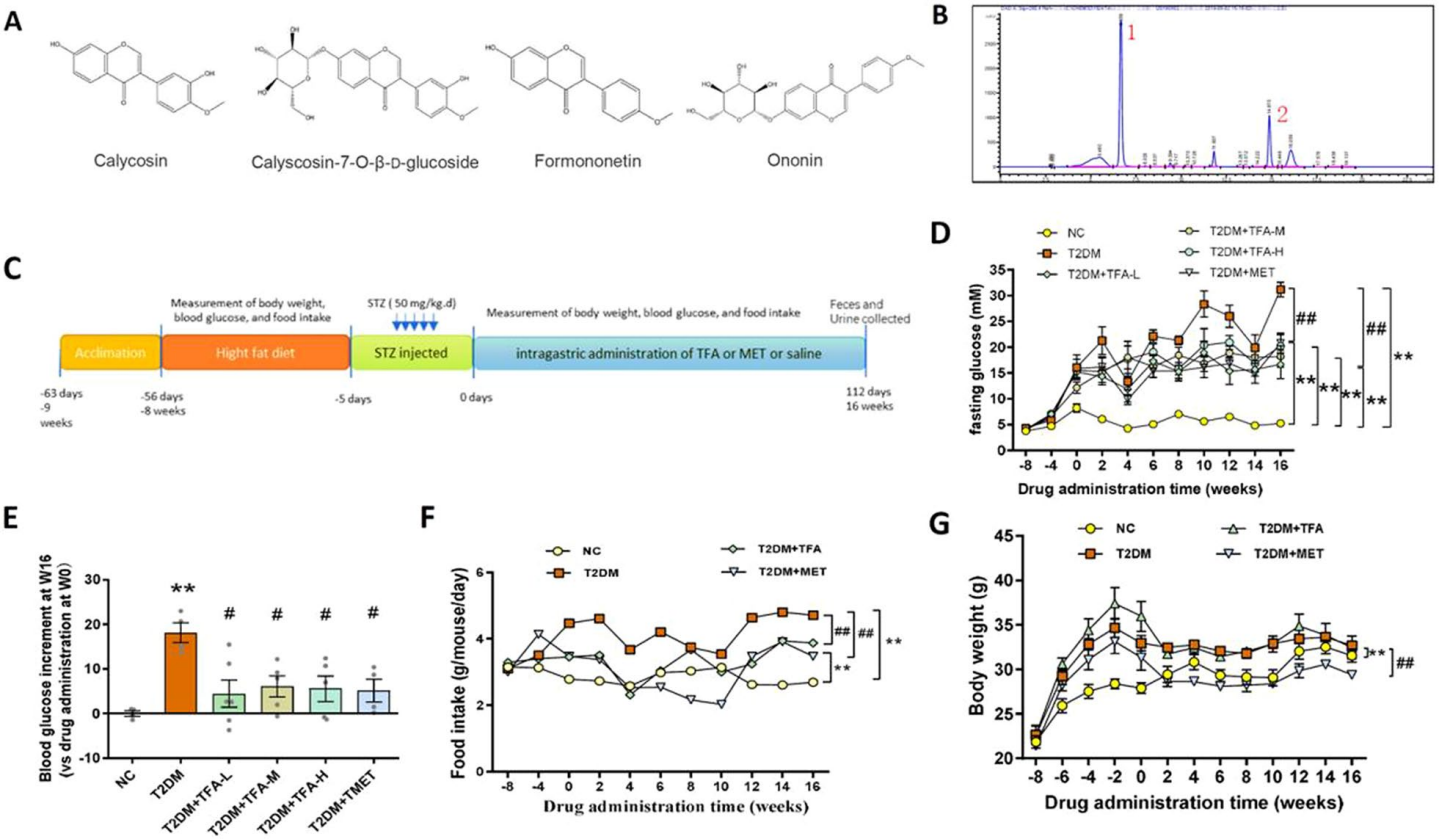
TFA alleviated blood glucose and body weight in high fat diet/STZ induced diabetic mice. **A** The main chemical components of flavonoids from *Astragalus membranaceus*. **B** HPLC chromatogram of total flavonoids from *Astragalus membranaceus* (the peak 1 is the isoflavone glycoside, and the peak 2 is the isoflavone). **C** Timeline depicting the treatment in each group. **D** Fasting glucose, **F** body weight, and **G** Food intake during the experiment. **E** Blood glucose decrement at Week 16. NC, natural control; T2DM: type 2 diabetes mellitus; TFA: total flavonoids of *Astragalus membranaceus*, TFA-L: 5 mg/kg d; TFA-L: 25 mg/kg d; TFA-H: 50 mg/kg d; MET: metformin, 0.15 g/kg d. *p < 0.05, **p < 0.01, vs. NC group; ^#^p < 0.05, ^##^p < 0.01, vs. T2DM group

To confirm quality of *total flavonoids from Astragalus membranaceus* (TFA), liquid chromatography was carried out. *Calycosin*, *Ononin*, *Calycosin-7-O-beta-d-glucoside*, and *Formononetin* are the main chemical components of flavonoids from *Astragalus membranaceus* (Fig. 1A); and we found main flavonoids were included in TFA: the peak 1 is the isoflavone glycoside, and the peak 2 is the isoflavone (Fig. 1B).

To induce T2DM, 8-week-old C57BL/6 J mice were fed with high fat diet for 8 weeks, and then injected with streptozotocin (STZ, 50 mg/kg d) for 5 days. Successful construction of T2DM was believed when the fasting blood glucose above 11.1 mmol/l or 120 min IPGTT ≥ 11.1 mmol/l. Diabetic mice were daily administrated with TFA or metformin as indicated for 16 weeks, and normal control mice (NC) were administrated with normal saline (Fig. 1C). As shown in Fig. 1D and E, TFA administration significantly decreased fasting blood glucose (Fig. 1D); at Week 16, decrement of fasting blood glucose in TFA-L group is more significant than that of T2DM, TFA-M, TFA-H or positive control (metformin) groups (Fig. 1E). More importantly, food intake was dramatically inhibited compared with T2DM group (Fig. 1F, p < 0.01). We did not find significant effect of TFA on reducing body weight in the present study (Fig. 1G).

### TFA ameliorated brain impairment and reduced Aβ aggregation

**Fig. 2:**
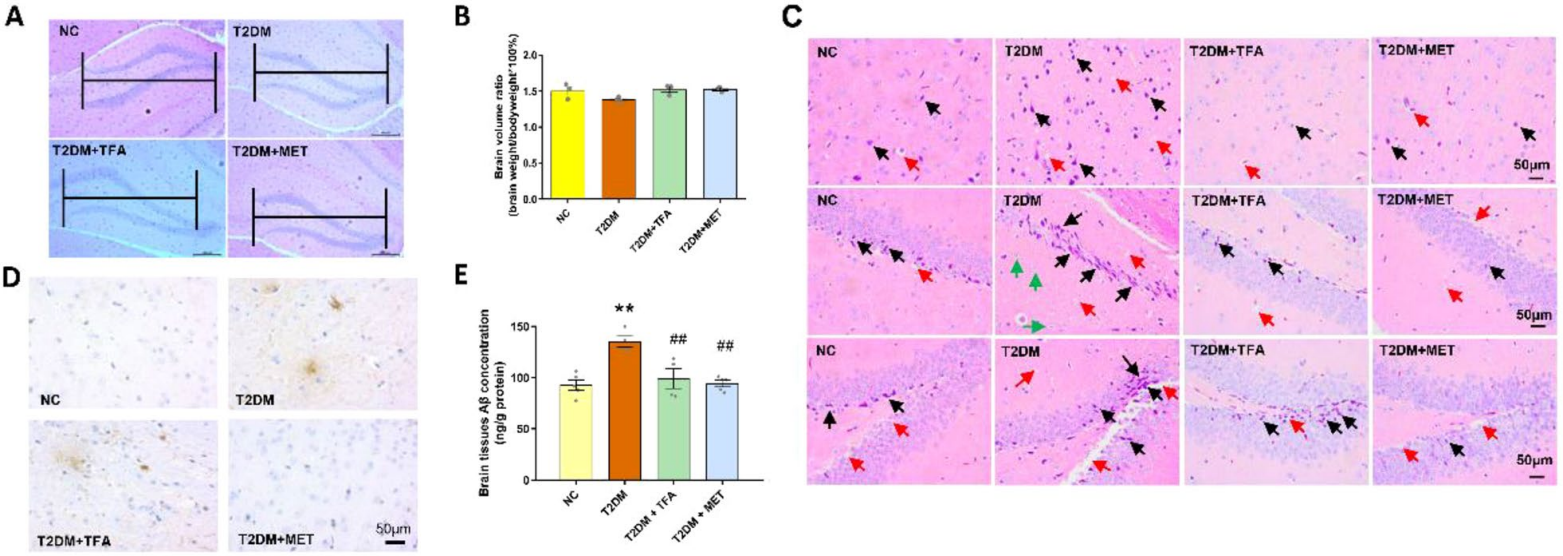
TFA ameliorated brain function and inhibited Aβ aggregated in type 2 diabetic mice. **A** H&E staining of mice hippocampus; **B** Brain volume ratio. **C** H&E staining of the brain (black arrow indicates the shrunken and hyperchromatic neurons; red arrow indicates the small empty bubble in the brain tissues; green arrow indicates the swollen, vacuolar neurons). **D** Immunohistochemistry and **E** ELISA detection of Aβ in the brain. *p < 0.05, **p < 0.01, vs. NC group; ^*#*^*p* < 0.05, ^*##*^*p* < 0.01, vs. T2DM group

Cognitive impairment is one of a common complication in T2DM. To firstly observe histological changes of the brain, H&E staining and immunohistochemistry were carried out. We found area of hippocampus as well as the whole volume of the brain in T2DM were decreased, the neurons in cortex and hippocampus in T2DM group were shown with loose structure and exhibited with cell body shrinking and vacuoles, and TFA administration ameliorated damage of the brain (Fig. 2A–C). Aβ aggregation within brain tissue has been recognized as cause and representation for cognitive disorders. By immunohistochemistry and ELISA (Fig. 2D and E), we found Aβ aggregation in the brain was significantly lowered compared with T2DM (*p* < 0.05), suggesting potential effects of TFA on ameliorating diabetic cognitive impairment.

### TFA improved synapse function and promoted BDNF expression

**Fig. 3:**
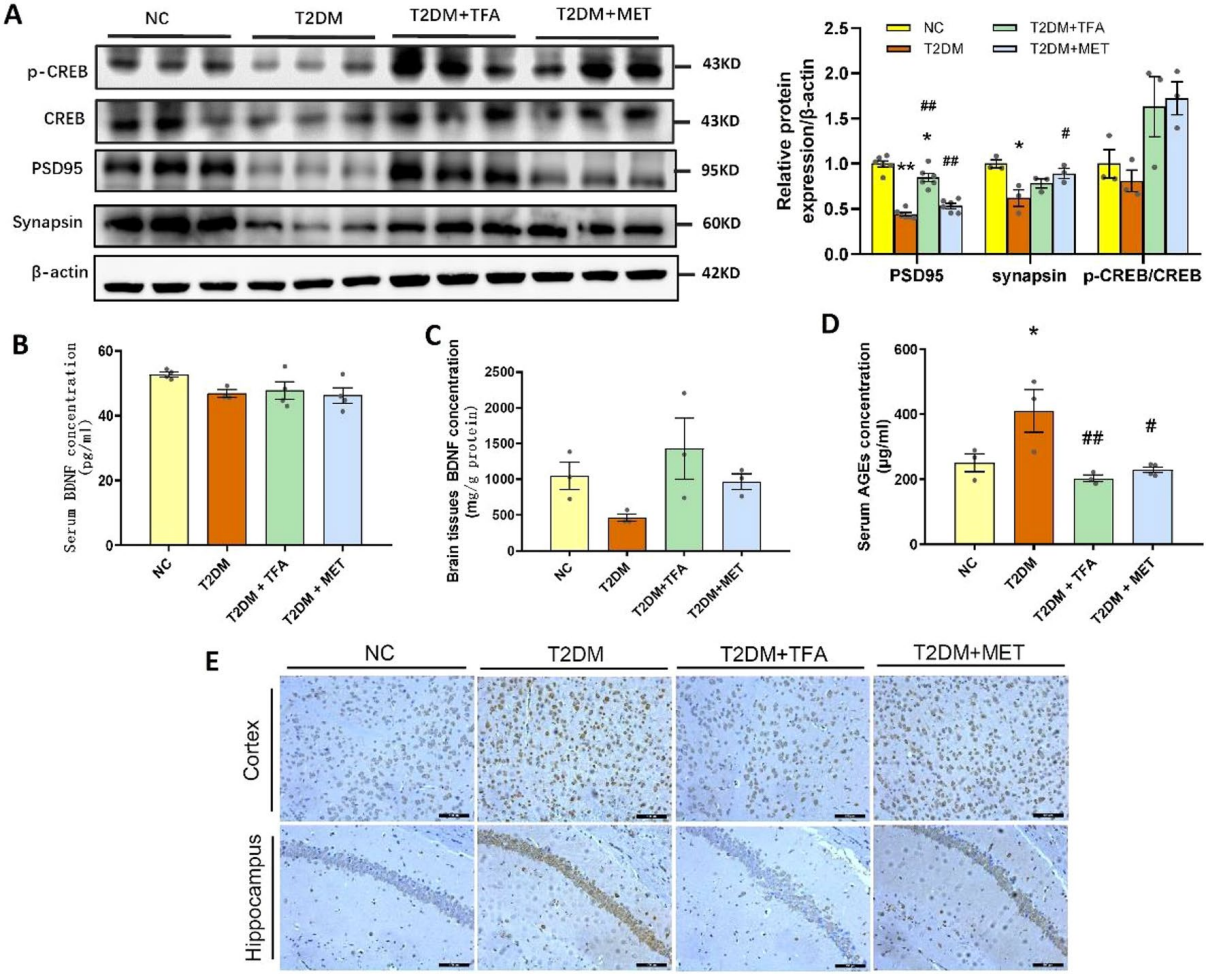
TFA improved synapse function in high fat diet/STZ induced diabetic mice. **A** Western Blots of the protein expression in hippocampus. **B** Concentration of BDNF in blood serum and **C** in brain tissue. **D** Concentration of AGEs in the serum. **E** Contents of GABA in brain cortex and hippocampus tested by immunohistochemistry. *p < 0.05, **p < 0.01, vs. NC group; ^*#*^*p* < 0.05, ^*##*^*p* < 0.01, vs. T2DM group

To further evaluate effects of TFA on diabetic cognitive impairment, expression of PSD95 and synapsin in hippocampus were detected by Western Blot to evaluate function of synapse. As observed in Fig. 3A, expression of PSD95 and synapsin in T2DM mice were dramatically decreased by as much as 40% (*p* < 0.05, vs NC), while TFA administration significantly up-regulated their expression (*p* < 0.01, vs T2DM).

BDNF is a neuro-peptide which is secreted by the autocrine manner and exerts effects on neuron grow and differentiation. In the present study, secretion of BDNF in serum and brain tissues was determined by ELISA. We observed in T2DM mice that BDNF was decreased in both serum and brain tissues, and TFA or MET administration significantly increased expression of BDNF in the brain (Fig. 3B and C). Further study demonstrated that the up-regulation of BDNF was via its upstream signaling pathway, CREB, as expression and activation of p-CREB was strengthened by TFA or MET in the hippocampus (Fig. 3A).

Advanced glycation end products (AGEs) has been believed as an inducer and promoter in diabetic complication, and its aggregation in the brain has been found to play a role in cognitive dysfunction [31, 32]. In the present study, we also observed TFA or MET significantly reduced level of AGEs in the brain (Fig. 3D). Latest research indicates that γ-aminobutyric acid (GABA) is associated with the development of neurodegenerative disease [33–35]. In our present study, we found that GABA was significantly increased in high fat and STZ induced diabetic mice brain, while was decreased to normal level after TFA treatment (Fig. 3E).

### TFA promoted mitochondrial biogenesis in the hippocampus

**Fig. 4:**
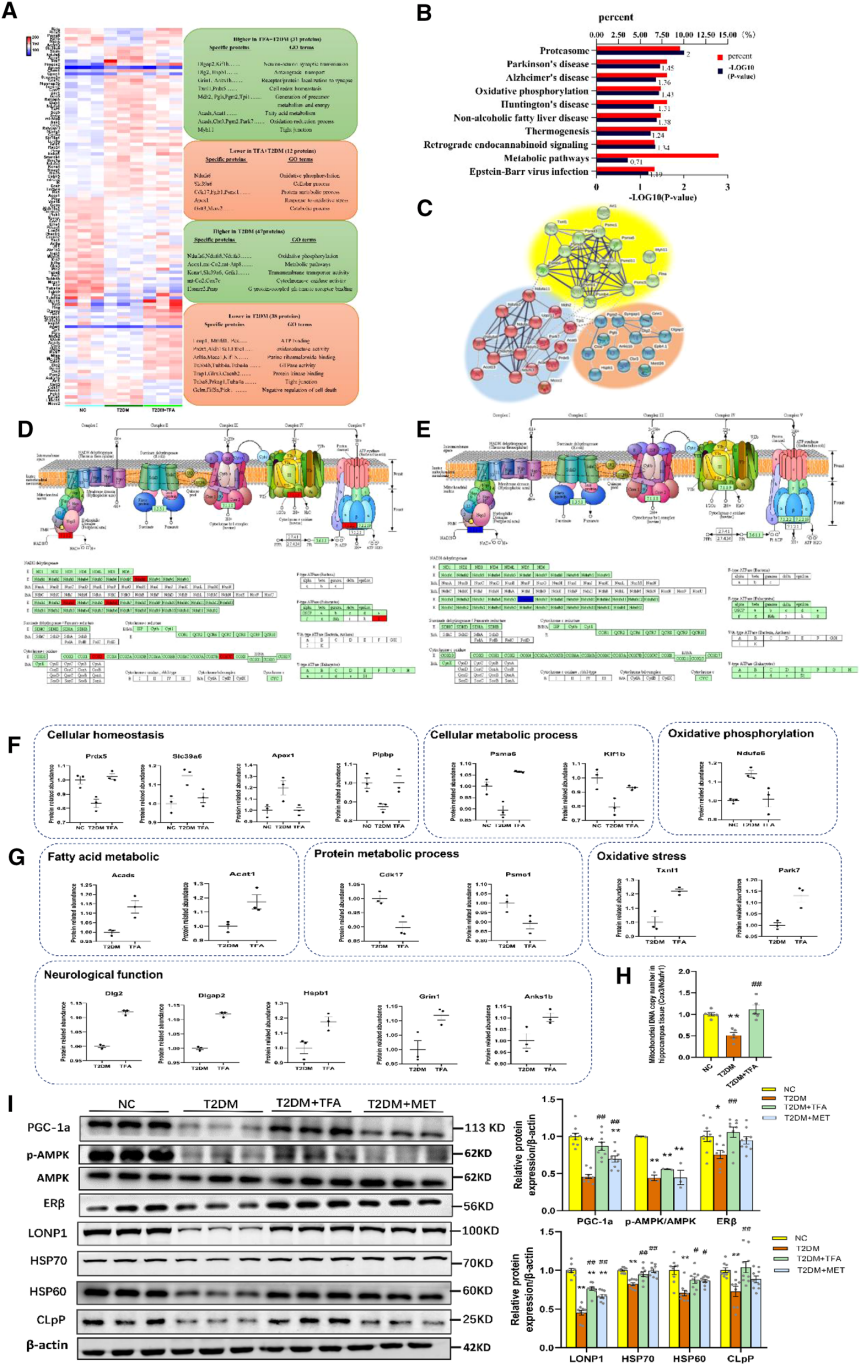
TFA promoted energy metabolism and mitochondrial biogenesis in the hippocampus of high fat diet/STZ induced diabetic mice. **A** Heatmap displaying the differentially expressed proteins in hippocampus between T2DM vs. NC group, and T2DM + TFA vs. T2DM group. **B** KEGG pathway analysis of differentially expressed proteins in T2DM + TFA vs. T2DM group. **C** Network diagram of protein interaction of differential expression protein in T2DM + TFA vs. T2DM group by STRING analysis. **D** KEGG analysis of oxidative phosphorylation (FDR-p < 0.05) of T2DM vs. NC, or **E** T2DM + TFA vs. T2DM based on differentially expressed proteins analysis, red column represents upregulation vs. NC group, blue column means downregulation vs. T2DM group. **F** Differentially expressed proteins in NC, T2DM, and T2DM + TFA group, and **G** T2DM + TFA vs. T2DM group. **H** Mitochondrial DNA levels in hippocampus tissue. **I** Western Blot of mitochondrial proteins in hippocampus tissue. *p < 0.05, **p < 0.01, vs. NC group; ^*#*^*p* < 0.05, ^*##*^*p* < 0.01, vs. T2DM group

To explore potential targets and mechanism of TFA in its modulated brain function, proteomics analysis of mice hippocampus tissue was performed. In the present study, a total of 3327 proteins were detected, 85 proteins were found to be differentially expressed in T2DM compared with NC group, and 43 were found to be differentially expressed in T2DM + TFA compared with T2DM group (Fig. 4A, p < 0.05). Among the 43 proteins, 31 was up-regulated while 12 were down-regulated. Through enriched GO terms analysis, we found the tight junction proteins were decreased in T2DM, while were increased by TFA administration (Fig. 4A); moreover, the energy metabolism was dysregulated in T2DM, and this was ameliorated after TFA treatment. KEGG pathway enrichment analysis showed that the differentially expressed proteins in T2DM + TFA compared with T2DM group were mainly related with metabolic, mitochondria dysfunction and neurodegeneration disease in the hippocampus (Fig. 4B). To further interpret the interaction among the 43 differentially expressed proteins, the protein–protein interaction (PPI) enrichment network analysis was performed by STRING. We found that the hub proteins were mainly enriched in oxidative phosphorylation, synaptic function and metabolic process (Fig. 4C).

In order to clearly show changes in electron transport chain (ETC), differential proteins were indicated in ETC pathway. We can clearly see that T2DM reduced ATP production by inhibiting the activity of NADH dehydrogenase and cytochrome c oxidase (Fig. 4D). While TFA treatment reserved this situation by increasing the activity of mitochondrial respiratory chain complex I (CxI) (Fig. 4E). The representative proteins in each biological process were show in Fig. 4F and G, which maybe the potential targets for TFA to against cognitive impairment in diabetes. Converging from results of proteomics and PPI analysis, we concluded that mitochondria might play a pivotal role in diabetic cognitive dysfunction. For this aim, level of mitochondrial DNA as well as the underlying mechanism was studied.

As the indicator of mitochondrial biogenesis, the mtDNA/nDNA ratio was firstly determined in hippocampus by Q-PCR. We found the copy number of mitochondrial DNA was significantly decreased in T2DM (*p* < 0.05, vs NC), and TFA administration significantly increased the copy number by over 2 times as compared with that in NC group (*p* < 0.01, vs T2DM and NC) (Fig. 4H).

To explore the underlying mechanism of TFA in mitochondrial biogenesis, expression and activation of intracellular signaling pathway proteins were determined by WB. As shown in Fig. 4I, TFA significantly reversed reduction of PGC-1α and p-AMPK/AMPK in diabetic mice (*p* < 0.01, vs T2DM); moreover, expression of mitochondria unfolded proteins including LONP1, CLPP, HSP60 and HSP70 were significantly increased by TFA to the level that was comparable to that of metformin (MET), suggesting TFA protected neuron function by promoting mitochondrial biogenesis and maintaining energy metabolic balance. Further study found effects of TFA was realized through estrogen receptor beta pathway, as its expression was elevated on TFA administration (Fig. 4I).

### TFA protected blood brain barrier (BBB) and gut barrier from diabetic damage

**Fig. 5:**
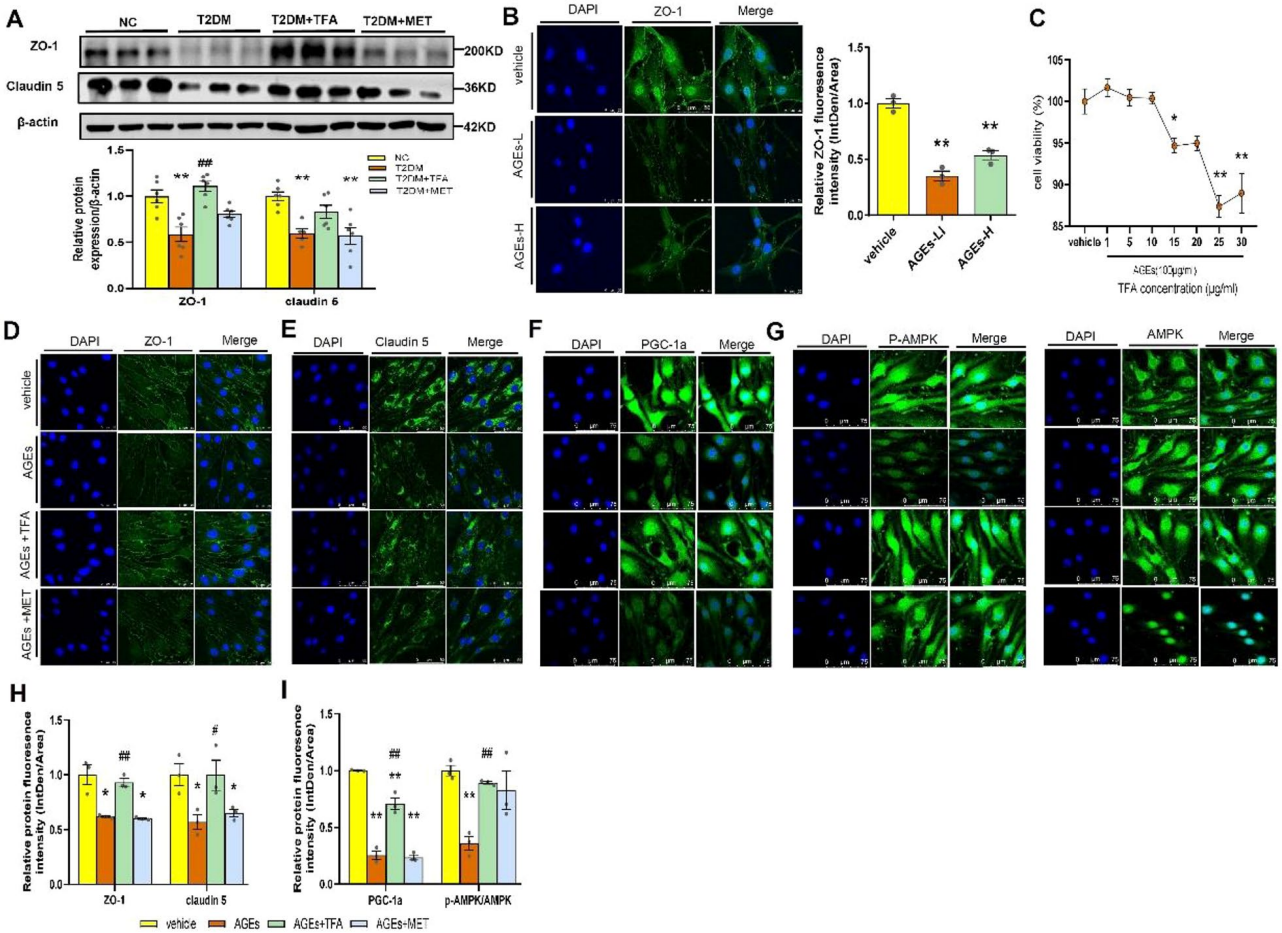
TFA increased the expression of conjunct proteins in brain hippocampus tissue. **A** Western Blot of ZO-1 and claudin 5. **B** Immunofluorescence of ZO-1 protein in Bend.3 cell induced by AGEs (AGEs-L: 100 μg/ml, AGEs-H: 200 μg/ml). **C** Cell viability tested by MTT. **D** ZO-1, **E** Claudin 5, **F** PGC-1α, **G** p-AMPK and AMPK proteins expression tested by immunofluorescence in Bend.3 cells; **H** and **I** relative protein fluorescence density analysis by Image J. AGEs: Advanced Glycation End products. *p < 0.05, **p < 0.01, vs. NC group or vehicle group; ^#^*p* < 0.05, ^##^*p* < 0.01, vs. T2DM group or AGEs group

Blood–brain barrier (BBB) plays a pivotal role in maintaining homeostasis of the brain. By proteomic analysis, we found tight-junction proteins in the brain were reduced in T2DM, while were elevated after TFA treatment (Fig. 4A). To elucidate effects of TFA on BBB, expression of ZO-1 and claudin 5 in hippocampus was determined by WB. As shown in Fig. 5A, TFA administration significantly increased expression of ZO-1 and claudin 5 in the hippocampus (*p* < 0.05, vs. T2DM). This was validated in the in vitro study in Bend.3 cells (Fig. 5B–E, and H) that TFA increased both cell viability and intracellular tight junction proteins’ proteins. We also observed that TFA significantly increased the protein expression and activation of PGC-1α and p-AMPK/AMPK in Bend.3 cells (Fig. 5F, G and I).

**Fig. 6:**
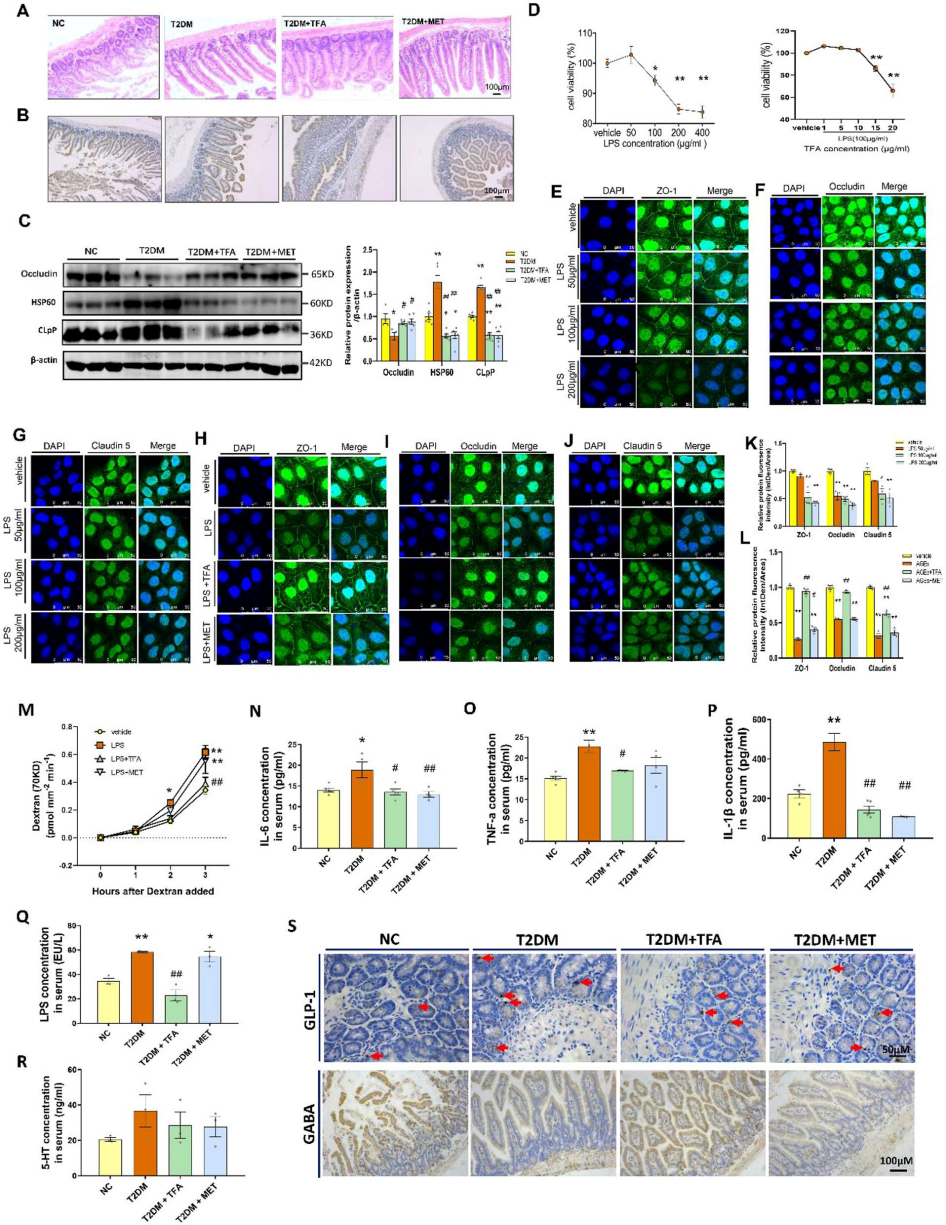
TFA ameliorated intestine barrier and decreased inflammation in high fat diet/STZ induced diabetic mice. **A** H&E staining of intestine tissue. **B** Occludin expression tested by immunohistochemistry. **C** Western Blot of proteins in intestine tissue. **D** MTT assay of CaCO_2_ cells viability under LPS or TFA treatment. Proteins expression assay of **E** ZO-1, **F** Occludin, and **G** Claudin 5 of CaCO_2_ cells under LPS treatment by immunofluorescence. Influence of TFA on the expression of **H** ZO-1, **I** Occludin, and **J** Claudin 5. **K** and **L** Protein fluorescence density analysis of for H-J by Image J. **m** Influence of TFA on the in vitro gut barrier model. The concentration of **N** IL-6, **O** TNF-α, **P** IL-1β, **Q** LPS, and **R** 5-HT in serum determined by ELISA. **S** Expression of GLP-1 and GABA in gut tested by immunohistochemistry (GLP-1 indicated by red arrow). LPS: Lipopolysaccharide. *p < 0.05, **p < 0.01, vs. NC group or vehicle group; ^*#*^*p* < 0.05, ^*##*^*p* < 0.01, vs. T2DM group or LPS group

Another barrier that preserves homeostasis of internal environment is gut barrier. It has been widely recognized that disruption of the integrity of gut barrier is closely associated with diabetic complications [36–38], as this will make gut-sourced endotoxin easier to enter into the body and induce a so called sustained low-grade inflammation. To this aim, we firstly observed morphological changes before and after TFA administration. As shown in Fig. 6A, the intestinal muscularis structure in NC group was tight, while this structure was strikingly changed in T2DM in that the villi density was sparse, muscularis of intestinal was relaxed, and the number of goblet cells was reduced; and TFA or MET administration improved the structure integrity. By immunohistochemistry and WB, we also assessed location as well as expression of tight junction proteins in the gut barrier. As observed in Fig. 6B and C, TFA or MET administration significantly elevated expression of tight junction protein occludin (*p* < 0.05, vs T2DM). The intestine barrier protective effect of TFA was further confirmed in the in vitro study of CaCO_2_ cells. With the treatment of TFA, the cell viability was elevated, protein expression of ZO-1, occludin, and claudin 5 were significantly increased and the gut barrier leakage was significantly dropped (Fig. 6D–M).

Tight junction of gut barrier relies on energy supply, thus mitochondrial function is important. To this end, expression of CLPP and HSP60, the core markers of mitochondrial unfold protein response (UPR^mt^), on gut lumen was determined by WB (Fig. 6C). We found CLPP and HSP60 were significantly up-regulated in T2DM mice (*p* < 0.01, vs NC), suggesting that UPR^mt^ was continuously activated in the gut of diabetic mice; and their expression was significantly down-regulated to the normal level after TFA or MET treatment.

To further evaluate functional integrity of gut barrier, levels of inflammatory cytokines in the serum were determined in diabetic mice. As shown in Fig. 6N–Q, contents of IL-6, TNF-α, IL-1β, and LPS were strikingly elevated in diabetic mice compared with control (*p* < 0.05, vs. NC), and this trend was significantly reversed by TFA administration. Interestingly, we also found reduction of 5-HT, which may inhibit nerve activation and induce emotion as well as cognition changes, on application of TFA, but no statistical significance was observed (Fig. 6R, TFA vs. T2DM). GLP-1, which is produced by *L* cells in intestinal mucosa, has been verified to possess effects on inhibiting the uptake of glucose [39], ameliorating T2DM, and improving memory performance [40, 41]. In the present study, we observed that GLP-1 was significantly elevated in intestine of T2DM mice, while was decreased after TFA administration (Fig. 6S, TFA vs. T2DM). Moreover, TFA or MET administration significantly ameliorated reduction of GABA in gut of the diabetic mice (Fig. 6S, TFA vs. T2DM).

It should be noted that LPS cannot be generated by the body itself, thus its decrement should attribute to: (1) gut integrity enhancement, or (2) its production reduction within gut lumen. In this sense, influence of TFA on abundance as well as composition of gut microbiota was assessed in the following study.

### TFA re-constructed composition of gut microbiota

**Fig. 7:**
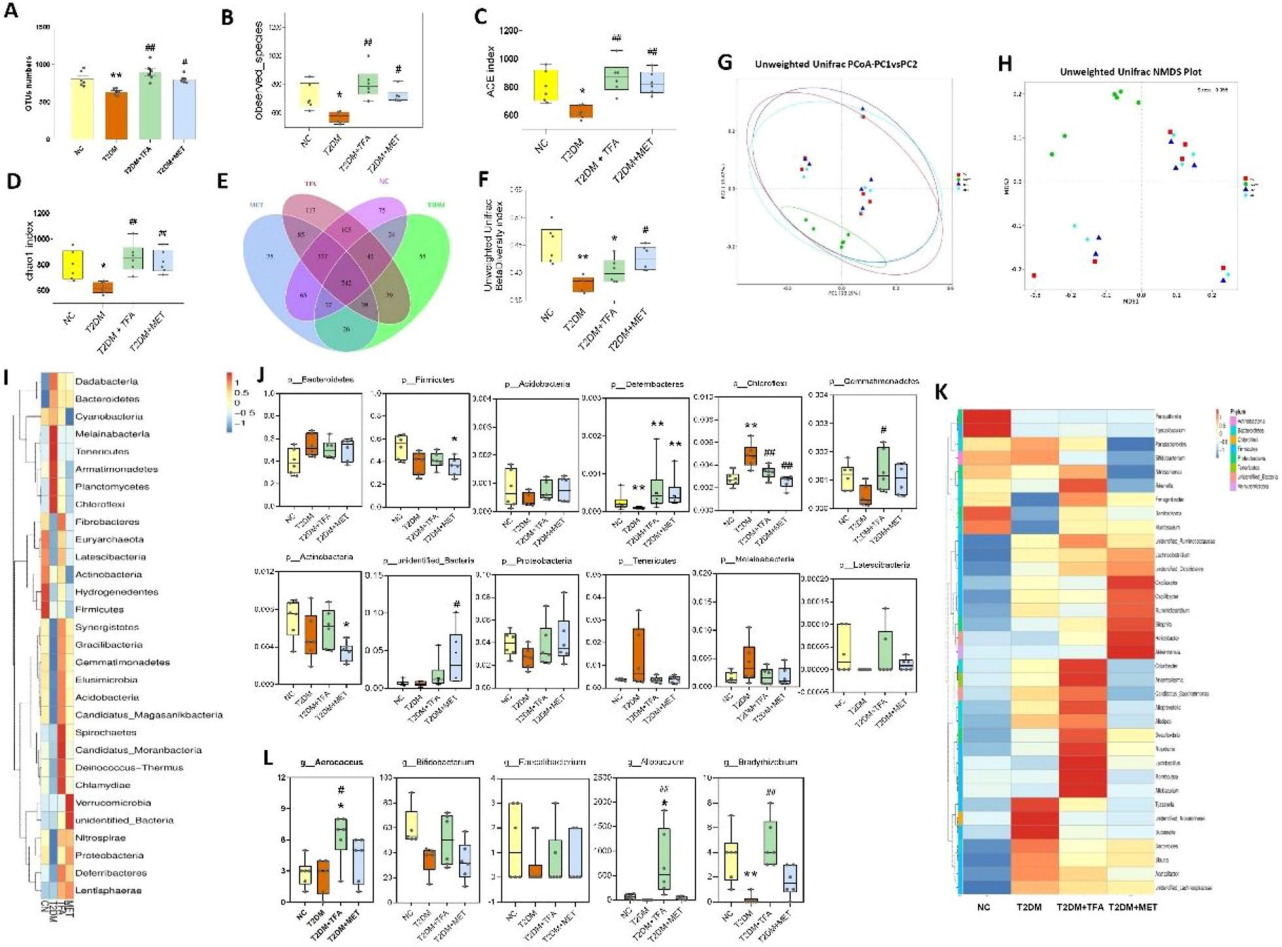
The analysis of gut microbiome and microbial metabolites. **A** Total number of observed OTUs. **B** Observed species analysis, **C** ACE assay, and **D** chao1 detection of gut microbiota in each group. **E** Venn Graph showing the common and unique OTUs among groups. **F** Unweighted Unifrac BetaDiversity index. **G** PCoA analysis. **H** NMDS analysis. **I** Heatmap showing the TOP30 Phylum species of in each group. **J** Relative abundance of differential phylum species was identified by an analysis of composition of microbiome (ANCOM) on the 16 weeks after treatment, Wilcoxon rank-sum test was adopted for comparisons between groups. **K** TOP35 differences genus species. **L** Differential Genus species. *p < 0.05, **p < 0.01, vs. NC group; ^*#*^*p* < 0.05, ^*##*^*p* < 0.01, vs. T2DM group

Intestinal microbes have been recognized to play an important role in ameliorating diabetes as well as cognitive impairment. Studies indicated that oral drug administration may influence disease development via modulating composition and metabolites of gut microbiota. To this end, gut microbiota as well as its metabolites were investigated in animals after 16 weeks’ oral administration with TFA. As shown in Fig. 7A, number of OTUs in diabetic mice was significantly decreased (*p* < 0.01, vs. NC), and TFA or MET treatment significantly increased abundance of OUTs to the normal levels. Analysis of observed species, ACE index and chao1 index suggested that administrating of TFA or MET was benefit for higher species richness in gut microbiota compared with that in T2DM group (Fig. 7B–D). Mice in TFA or MET group shared more common species with that in NC group (Fig. 7E–H). As depicted in Fig. 7I and J, although TFA treatment did not totally overturn changes of gut microbiota in diabetic mice, abundance of probiotics, such as butyrate-producing *p_Acidobacteria*, *p_Proteobacteria*, *p_Gemmatimonadetes*, *p_Deferribacteres*, *p_unidentified_Bacteria* and *p_Latescibacteria* was dramatically increased, while microbiota including *p_Bacteroidetes*, *p_Tenericutes*, *p_Melainabacteria* and *p_Chloroflexi* was decreased (Fig. 7J). Further study by genus species analysis (Fig. 7K and L) showed that TFA administration significantly increased abundance of *g_Aerococcus*, *g_Bifidobacterium*, and *g_Faecalibacterium* et al. Converging evidence suggested that TFA application increased abundance as well as diversity of gut microbiota; more importantly, abundance of butyrate-producing bacteria was significantly elevated, suggesting metabolites of microbiota (e.g., butyrate) may play an important role in it.

**Fig. 8:**
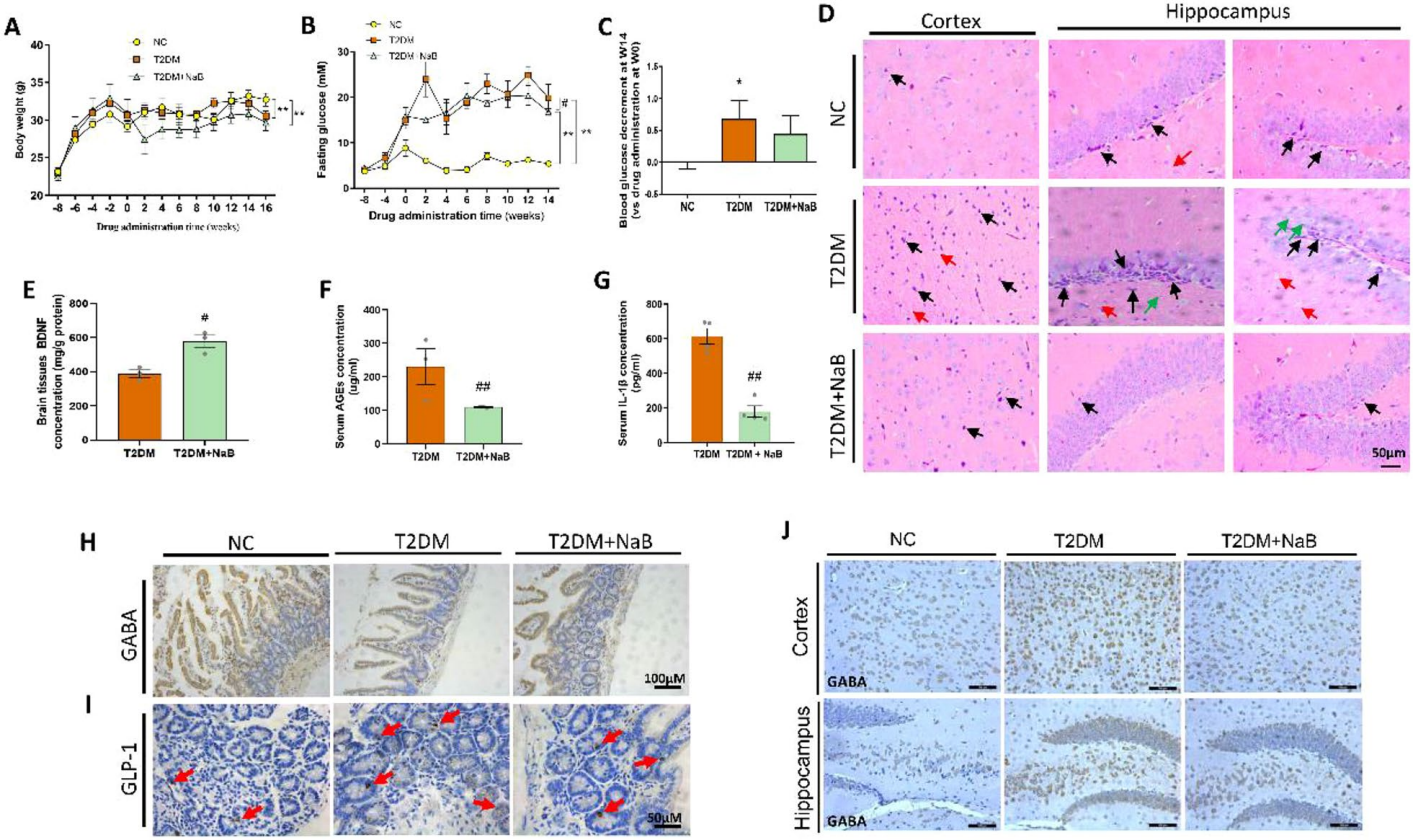
The effects of NaB on brain function in STZ induced diabetic mice. **A** Body weight, and **B** Fasting blood glucose in mice during the experiment. **C** Fasting blood glucose decrement at Week 14. **D** H&E staining of mice brain (black arrow indicates the atrophic and deep staining of neurons; red arrow indicates the loose structure of brain tissues; and the swollen, vacuolar neurons were indicated by green arrow). ELISA assay for concentration of **E** BDNF in brain tissue, and **F** AGEs and **G** IL-1β in serum. **H** IHC analysis of GABA and **I** GLP-1 in intestine tissues (red arrow indicates the GLP-1). **J** Expression of GABA in brain cortex or hippocampus by immunohistochemical analysis. *p < 0.05, **p < 0.01, vs. NC group; ^*#*^*p* < 0.05, ^*##*^*p* < 0.01, vs. T2DM group

To demonstrate the above hypothesis, STZ-induced diabetic mice were orally administrated with sodium butyrate (NaB), and neuron changes was studied. As shown in Fig. 8A–C, NaB oral administration slightly reduced body weight in diabetic mice, but no statistical significance was found; however, NaB significantly reduced fasting blood glucose level as compared with T2DM group. Brain pathology study by H&E staining indicated that degeneration such as neuron shrinkage (black arrow) and swelling (green arrow) were more common in diabetic brain (Fig. 8D), while NaB reversed this damage.

To preliminary explore mechanism of NaB in diabetic brain injury, we determined level of BDNF in brain by ELISA and found its expression was significantly elevated by TFA treatment (Fig. 8E); moreover, levels of AGEs and IL-1β were strikingly reduced (*p* < 0.01, vs. T2DM) (Fig. 8F and G). Interestingly, we found GABA was decreased in the intestine tissue, while increased in brain tissue in T2DM mice; and after NaB treatment, the content of GABA was returned to normal level (Fig. 8H–J). We also observed that the increased expression of GLP-1 within gut lumen was reduced by 16 weeks’ oral administration with NaB (Fig. 8J).

### TFA ameliorated mitochondrial dysfunction in AGEs-induced HT22 cells

**Fig. 9:**
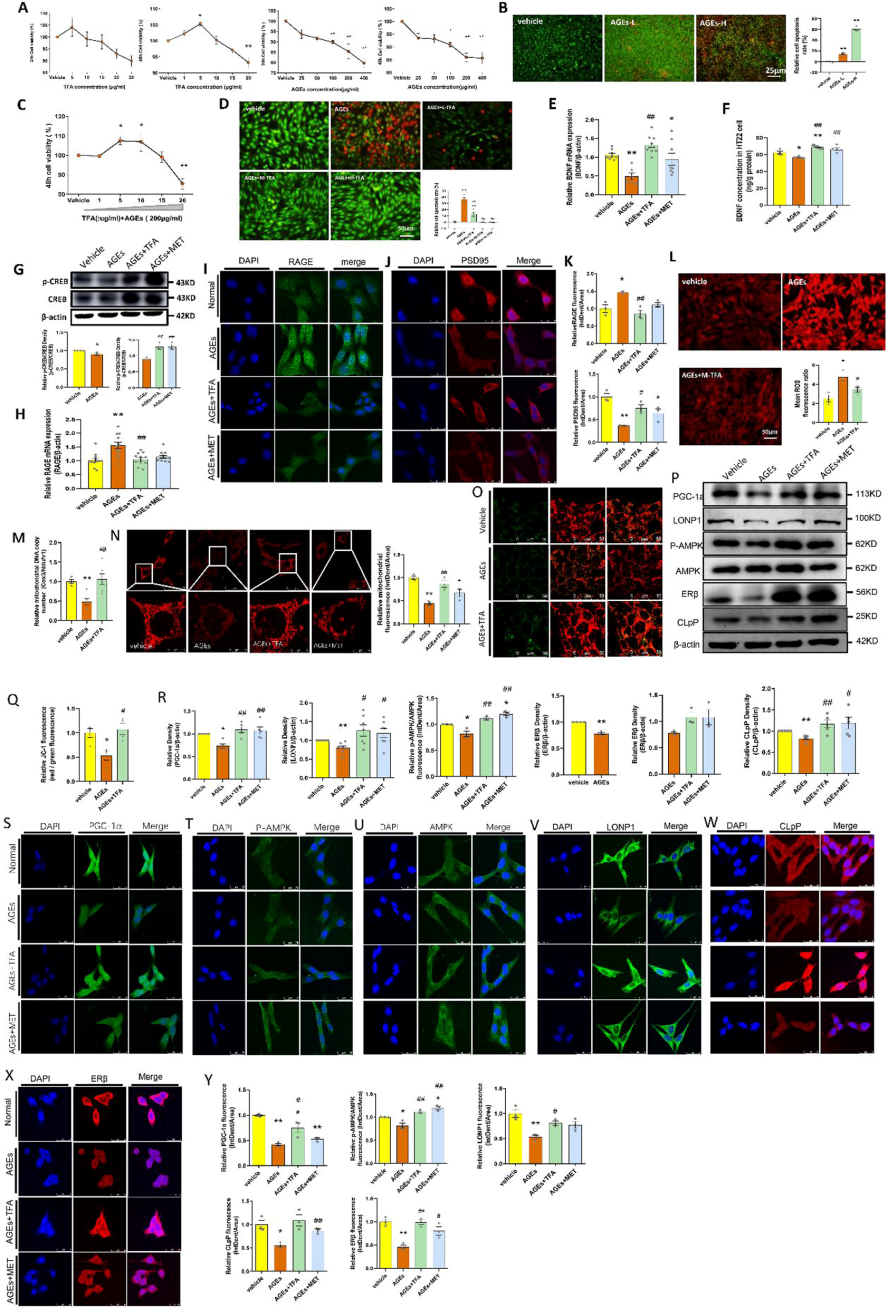
TFA ameliorated mitochondrial function in HT22 cells. **A** Cell viability analysis of HT22 cells under AGEs or TFA for 24 h or 48 h tested by MTT. **B** Apoptosis influence of HT22 cells under AGEs tested by AO/EB staining observed under a fluorescence microscopy (magnification: 200), AGEs-L:100 μg/ml, AGEs-H:200 μg/ml. **C** HT-22 cells viability by MTT assay. **D** AO/EB staining of HT22 cells under TFA or AGEs treatment, the image was observed by fluorescence microscopy (magnification: 400), L-TFA: 1 μg/ml, M-TFA: 5 μg/ml, H-TFA: 10 μg/ml. **E** mRNA and **F** protein expression of BDNF in HT22 cells. **G** Expression of p-CREB determined by WB. **H** RAGE mRNA expression tested by Q-PCR. Protein expression of **I** RAGE and **J** PSD95 observed by confocal microscopy (magnification: 1200), **K** density of RAGE and PSD95 was compared based on data of **I** and **J**. **L** Dihydroethidium staining analysis of ROS observed by fluorescence microscopy (magnification: 400) and the intracellular fluorescence intensity was analyzed by Image J. **M** Mitochondrial copy number tested by Q-PCR. **N** The content of mitochondrial tested by Mitochondrial Tracker™ deep Red FM (magnification: 1200 and 3200). **O** Mitochondrial membrane potential assay by JC-1 staining observed under confocal microscope (magnification: 1200). **P** The proteins expression of PGC-1α, LONP1, p-AMPK/AMPK, and CLpP by WB. **Q** Red/Green fluorescence ratio analysis was tested for data of **O**. **R** Gray density analysis of data **P** was analysis by Image J. Immunofluorescence analysis of **S** PGC-1α, **T** p-AMPK, **U** AMPK, **V** LONP1, **W** CLpP, and **X** ERβ proteins observed by a laser scanning confocal microscope (magnification: 1200), and **Y** the protein fluorescence density was analyzed by Image J software. *p < 0.05, **p < 0.01, vs. vehicle group; *#p* < 0.05, *##p* < 0.01, vs. AGEs group

AGEs has been widely recognized to be an inducer and promoter in diabetic complications [42]. To further investigate mechanism of TFA on protecting brain function, HT22 cell line was applied. We firstly incubated HT22 cells with different concentrations of TFA for 24 or 48 h (Fig. 9A), and found TFA at 5 μg/ml has most significant effects on increasing cell viability. While AGEs inhibited cell viability in a concentration-dependent manner (Fig. 9A). Converging with published reports, we choose 200 μg/ml of AGEs to induce cell damage. We found when the cells were co-incubated with 200 μg/ml AGEs and 5 μg/ml TFA, the viability was highest (Fig. 9C). In order to determine the optimal concentration of AGEs and TFA, the apoptosis test was performed by acridine orange (AO)/ethidium bromide (EB) staining. We observed that the cell apoptosis was significantly increased under 200 μg/ml AGEs incubation, while reserved to normal levels by 5 μg/ml or 10 μg/ml of TFA after 48 h treatment (p < 0.01, vs. AGEs) (Fig. 9B and D).

The influence of TFA on the secret of BDNF was verified in HT22 cells by ELISA and Q-PCR. We observed that the level of BDNF was significantly down-regulated under AGEs administration, while increased by TFA; further studies suggested this was due to the increased expression and activation of CREB (Fig. 9E–G). RAGE, as a receptor of AGEs, has been considered as a contributor to causes of cognitive decline. In the present study, the levels of RAGE were tested by Q-PCR and immunofluorescence. We found RAGE was significantly decreased by TFA (*p* < 0.01, vs. AGEs) (Fig. 9H, I and K). Moreover, TFA increased the protein expression of PSD95 (*p* < 0.05, vs. AGEs) (Fig. 9J and K). Proteomic analysis in this study found that the pathogenesis of diabetic cognitive impairment is closely related to mitochondrial dysfunction (Fig. 4A and D). Based on this finding, ROS as the indicator of mitochondrial dysfunction and repair was tested by Dihydroethidium staining. We found 5 μg/ml TFA decreased the content of AGEs-induced ROS in HT22 cells (*p* < 0.05, vs AGEs) (Fig. 9L). The mitochondrial number (Fig. 9M and N), the mitochondria membrane potential (Fig. 9O and Q), as well as the activity of antioxidant enzyme (Table 2) were dramatically elevated on TFA application. Mechanism investigation suggested that TFA modulated mitochondrial function via modulating its biosynthesis and energy metabolism (Fig. 9P, and R–W), and estrogen receptor beta may participate in this modulation (Fig. 9X).

**Table 2:** Activity of enzymes related to oxidative stress (n = 5)

Groups	Vehicle	AGEs	AGEs + TFA
SOD (mU/mg prot)	230.9 ± 0.2240	217.9 ± 0.2960**	223.7 ± 0.3405**^##^
MDA (nmol/mg prot)	0.8380 ± 0.006907	1.112 ± 0.01516**	1.036 ± 0.01266**^##^

AGEs, Advanced glycation end products; TFA, total flavonoids of *Astragalus membranaceus*; SOD, Superoxide Dismutase; MDA, Malondialdehyde

^**^p < 0.01, vs. Vehicle

^##^p < 0.01, vs. AGEs

## Discussion

Cognitive decline is a serious complication of diabetes. Although much efforts have been paid, limited progress is achieved. In the present study, we found and demonstrated Flavonoids from *Astragalus membranaceus* can help to ameliorate progression of diabetic cognition decline, and gut-brain axis may play a pivotal role in it.

*Astragalus membranaceus* (*Huang Qi* in Chinese) has been used as a herbal medicine to preserve health and treat a series of chronic diseases including diabetes for hundreds of years. Flavonoids are one of a main component of *Astragalus membranaceus*. Flavonoids have low bioavailability, only 10% can be absorbed by the intestine, and the amount of flavonoids that across blood brain barrier and enter into the brain is even less. The low biological utilization of flavonoids significantly prohibits research and development of this product to some extent [43, 44]. However, studies have proved that flavonoids can be metabolized by intestinal flora and become more bioactive flavonoids derivatives [45]. Previously, some reports indicated that flavonoids from *Astragalus* can ameliorate diabetes-induced brain dysfunction [17, 18]. With the understanding of the pivotal role of gut in preserving health and modulating drug-mediated therapeutic effects, we hypothesized that flavonoids from *Astragalus membranaceus* (TFA) may function via gut-brain axis. For this aim, we designed this study.

In high-fat and STZ-induced diabetic mice, we found TFA administration preserved structure of hippocampus and reduced deposition of Aβ in the brain. Synaptic function dysregulation has been recognized to be correlated with cognitive dysfunction and Alzheimer’s disease (AD) [46, 47]. In the present study, we found TFA treatment stimulated the CREB/BDNF signaling pathway and increased the production of the neurotrophic factor BDNF. Consistently, the upregulation of PSD95 and synapsin in the hippocampus after TFA treatment further demonstrated the benefits of TFA on synaptic function. These data provided direct evidence that TFA is benefit to preserve brain function under diabetes settings.

The survival of nerve cells, the transmission of neurotransmitters, and the maintenance of synaptic function are depended on energy produced by the mitochondria. Mitochondrial unfolded protein responses (UPR^mt^), which involves in oxidative phosphorylation [48], are important processes for maintaining mitochondrial function, linking aging to neurodegenerative diseases [49, 50]. LONP1 and CLpP are the two main proteases to regulate UPR^mt^. Previous studies demonstrated that LONP1 regulates the protein expression of mitochondrial complex IV and V and then decrease ATP generation [51], and CLpP inhibits mitochondrial oxidative phosphorylation by regulating mitochondrial complex I [48]. Here, we found that TFA significantly upregulated expressions of LONP1 and CLpP and improved the generation of ATP in hippocampus of the diabetic mice; moreover, energy metabolism and mitochondrial biogenesis-related pathway AMPK/PGC1α was involved in this regulation.

It is believed that mitochondrial dysfunction of neurons can activate the mitochondrial unfolded protein response of intestine and promote the secretion of metabolic signals in response to misfolded protein stress [52]. We found in the present study that the significantly increased content of GABA in cortex and hippocampus of the diabetic mice were strongly related to the abundance of *Bacteroides* and *Parabacteroides* within gut lumen (Figs. 3E and 7J), which is consistent with previous report [53, 54]. On the other hand, TFA administration reduced expression of GABA in hippocampus, re-balanced composition of gut microbiota, promoted BBB integrity, reduced levels of inflammatory cytokines and AGEs, and preserved homeostasis of internal environment in the brain. This suggested that there was a strong correlation of gut microbiota and brain function [4, 55].

Previous studies have shown that diabetes will reduce the diversity of gut microbes [56, 57]. In the present study, butyrate-producing bacteria including *p_Acidobacteria*, *g_Roseburia*, *g_Allobaculum*, and *g_Faecalibacterium* et al. were found to be significantly enriched after TFA administration, suggesting involvement of these bacteria in diabetes. Reports demonstrated that these bacteria are strongly related to diabetic complications [58–62]. In a most recent clinical study, Mirjam and colleagues found that lower abundance of butyrate-producing bacteria was related with a compromised cognitive function [63]. To test direct involvement of butyrate in diabetic brain dysfunction, diabetic mice were orally administrated with sodium butyrate in the present study; we found the brain structure was improved, expression of BDNF was increased while GABA was decreased in both cortex and the hippocampus. Studies have shown that butyric acid can cross blood brain barrier [64], inhibit NOX2 and reduce Aβ aggregation [65], and improve memory dysfunction in late advanced stage of AD mice [66]. Previously, we found protective effects of both oral [67] or intraperitoneal [68] administration of sodium on metabolism of diabetic mice. We postulated that modulation of energy metabolism might play a fundamental role in its effects on brain function.

Generation and aggregation of AGEs has been recognized to be an inducer and processer in diabetic neurological dysfunction [20, 69, 70]. Abundant studies have demonstrated that AGEs is closely related with inflammation and high blood glucose memory, and it has been regarded as a drug target in diabetic complications. High dietary AGEs is reported to be associated with Aβ deposition in hippocampus and poor learning ability in AD mice [71]. Generation of AGEs is always along with production of ROS. Research from Karlsson and colleagues showed that *Roseburia* and *Lactobacillus* species involve in antioxidant processes [72]. Previously, we found butyrate supplement could inhibit ROS generation and protect mitochondria function in HepG2 cells via PGC1α/AMPK pathway [68]. In the present study, we further observed oral TFA administration significantly increased abundance of *Roseburia* and *Lactobacillus* within gut lumen, the gut integrity was enhanced, production of inflammatory cytokines was decreased, and finally serum AGEs was reduced. We believe reduction of gut-sourced generation and absorption of AGEs contributed to decelerated deposition of Aβ in the brain.

## Conclusion

**Fig. 10:**
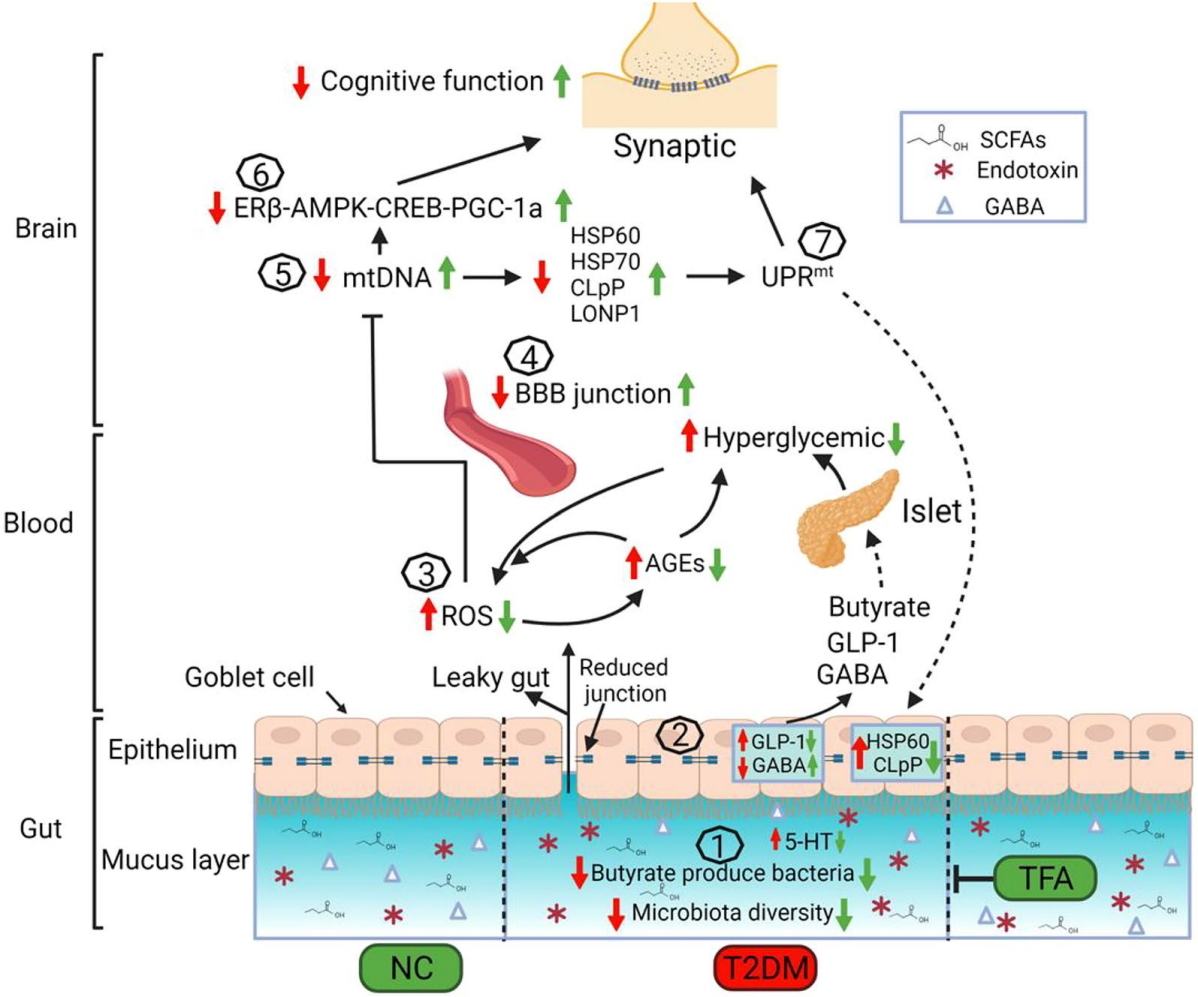
Role of gut-brain axis in TFA ameliorated diabetic brain dysfunction. Under normal settings, gut microbiota was in a stable state and regulate the secretion of neuropeptide (GABA, GLP-1, 5-HT, et al.). Long-term high-fat diet will change the homeostasis of gut microbiota and contribute to cognition dysfunction (steps **1**–**7**). **1** TFA increases the gut microbiota diversity and the number of functional bacteria, **2** restores intestinal homeostasis, **3** reduces oxidative stress, **4** protects the blood–brain barrier and **5** synaptic mitochondrial DNA, thereby **6** and **7** protects brain from damage

In conclusion, this study demonstrated that oral administration with flavonoids from *Astragalus membranaceus* reduced deposition of Aβ in hippocampus and ameliorated brain impairment, and its modulation on gut-brain axis plays a pivotal (Fig. 10). Our present study presented a novel finding of TFA on brain function, and provided an alternative solution on preventing and treating diabetic cognition impairment.

## Data Availability

All data used in the presented study can get from the corresponding author upon request.

## Abbreviations

AD: Alzheimer’s disease
AGEs: Advanced glycation end products
Aβ: Amyloid β-protein
BBB: Blood brain barrier
BDNF: Brain derived neurotrophic factor
CLPP: Caseinolytic protease
ERβ: Estrogen receptor β
GABA: γ-Aminobutyric acid
GLP-1: Glucagon-like peptide 1
HFD: High-fat-diet
HSP60: Heat shock proteins 60
HSP70: Heat shock proteins 70
H&E: Hematoxylin–eosin
IF: Immunofluorescence
IHC: Immunohistochemistry
IL-1β: Interleukin-1β
IL-6: Interleukin-6
IPGTT: Intraperitoneal glucose tolerance test
LONP1: Lon protease
LPS: Lipopolysaccharide
MET: Metformin
PSD95: Postsynaptic density protein 95
RAGE: Advanced glycosylation end product-specific receptor
TFA: Total flavonoids from *Astragalus membranaceus*
TFA-H: High-dose TFA
TFA-L: Low-dose TFA
TFA-M: Medium-dose TFA
TNF-α: Tumor necrosis factor-α
T2DM: Type 2 diabetes mellitus
UPR^mt^: Mitochondrial unfold protein response
WB: Western blot
5-HT: 5-Hydroxytryptamine/serotonin
